# Getting ready for an emotion: specific premotor brain activities for self-administered emotional pictures

**DOI:** 10.3389/fnbeh.2014.00197

**Published:** 2014-05-28

**Authors:** Rinaldo L. Perri, Marika Berchicci, Giuliana Lucci, Rocco L. Cimmino, Annalisa Bello, Francesco Di Russo

**Affiliations:** ^1^Department of Human Movement, Social and Health Sciences, University of Rome “Foro Italico,”Rome, Italy; ^2^Department of Psychology, University of Rome “La Sapienza,”Rome, Italy; ^3^Neuropsychology Unit, IRCCS Santa Lucia FoundationRome, Italy

**Keywords:** emotions, expectancy, Movement Related Cortical Potentials (MRCPs), Event Related Potentials (ERPs), Late Positive Potentials (LPPs)

## Abstract

Emotional perception has been extensively studied, but only a few studies have investigated the brain activity preceding exposure to emotional stimuli, especially when they are triggered by the subject himself. Here, we sought to investigate the emotional expectancy by means of movement related cortical potentials (MRCPs) in a self-paced task, in which the subjects begin the affective experience by pressing a key. In this experiment, participants had to alternatively press two keys to concomitantly display positive, negative, neutral, and scrambled images extracted from the International Affective Pictures System (IAPS). Each key press corresponded to a specific emotional category, and the experimenter communicated the coupling before each trial so that the subjects always knew the valence of the forthcoming picture. The main results of the present study included a bilateral positive activity in prefrontal areas during expectancy of more arousing pictures (positive and negative) and an early and sustained positivity over occipital areas, especially during negative expectancy. In addition, we observed more pronounced and anteriorly distributed Late Positive Potential (LPPs) components in the emotional conditions. In conclusion, these results show that emotional expectancy can influence brain activity in both motor preparation and stimulus perception, suggesting enhanced pre-processing in the to-be-stimulated areas. We propose that before a predictable emotional stimulus, both appetitive and defensive motivational systems act to facilitate the forthcoming processing of survival-relevant contents by means of an enhancement of attention toward more arousing pictures.

## Introduction

The early identification of emotionally relevant information is critical for survival (Darwin, 1872), and the anticipation of future affective events is a crucial skill of the human brain because it allows people to prepare the most adaptive response. Emotional expectancy entails multiple cognitive and motor processes, such as emotional regulation, retrieval of prior relevant events and preparation of the appropriate behavioral responses. In experimental neuroscience, it is important to distinguish anticipation from preparation. Anticipation consists in passively waiting for the stimulus, and it is a perception-oriented stage of the expectancy process, whereas preparation is a more motor-related stage during which the motor system is getting ready for motor execution (van Boxtel and Böcker, 2004).

Electroencephalographic (EEG) studies revealed three slow cortical potentials related to the expectancy and preparation processes: the Movement Related Cortical Potentials (MRCPs), the Contingent Negative Variation (CNV), and the Stimulus Preceding Negativity (SPN). The MRCPs are elicited by any voluntary movement and are interpreted as an index of the progressive cortical excitability necessary to prepare and execute movements. Among the MRCPs, one of the most studied is the Bereitschaftspotential (BP), which is a slow negative activity that for self-paced movements, begins approximately 2–3 s before the movement onset and reflects motor preparation (Shibasaki and Hallet, 2006) in premotor and motor brain areas but also anticipation processes, such as stimulus timing evaluation (Berchicci et al., 2012a, 2013, 2014; Di Russo et al., 2013a,b) and awareness of the consequences produced by the act (Di Russo et al., 2005a; Bozzacchi et al., 2012a,b) in prefrontal and posterior parietal areas. Conversely, the CNV and SPN are slow negative potentials reflecting the orientation to the upcoming stimulus presentation; thus, they can be related to the abovementioned perception-oriented process of the expectancy (for a review see van Boxtel and Böcker, 2004). Few studies have investigated emotions throughout the CNV (e.g., Mercado et al., 2007) and SPN (e.g., Takeuchi et al., 2005) waves, and they have partially explained the neurophysiological mechanisms underlying the expectancy of predictable emotions, but none of the available research investigated the effect of the emotional expectancy by means of MRCPs analysis.

The enhancement of the CNV and SPN potentials was described as arousal-dependent for pharmacological (Kopell et al., 1974), clinical (Wessa and Flor, 2007), and healthy subjects studies (Böcker et al., 2001; Takeuchi et al., 2005; Poli et al., 2007).

Nonetheless, other authors reported an opposite emotion-dependent modulation, showing a reduced CNV amplitude during the anticipation of unpleasant stimuli (Casement et al., 2008; Moser et al., 2009; Hart et al., 2012).

The conflicting results reveal that the role of emotions in anticipatory processes is still a matter of debate. The explanation for the inconsistent findings might be at least two fold: (1) the SPN is not a unitary phenomenon but a class of anticipatory responses, some of which are motivationally oriented, fear-related or subjectively relevance dependent (van Boxtel and Böcker, 2004); (2) the CNV-SPN paradigms did not control all of the methodological variables, such as the timing, the motor response after the stimulus or the presence of feedback.

The modulation of emotional expectancy has also been investigated by means of functional magnetic resonance imaging (fMRI); in visual cued tasks, an increased activation was observed in the left dorsolateral and medial prefrontal cortex (MPFC) during positive expectancy (Ueda et al., 2003) and in the right dorsolateral prefrontal cortex (DLPFC) and orbitofrontal and anterior cingulate cortices during negative respect to neutral expectancy (Davidson and Irwin, 1999; Nitschke et al., 2006). In addition, a few works recording peripheral indexes demonstrated that movement speed and force production varied as a function of emotional valence (Coombes et al., 2009); in particular, the negative affective state activates the defensive circuitry (Coombes et al., 2005, 2006), suggesting the involvement of motor-related central processes (Coombes et al., 2007).

Considering the low temporal resolution of fMRI and the before mentioned CNV-SPN methodological limitations, we sought to investigate the emotional expectancy by means of high-density EEG recording and MRCPs analysis. The main goal of this study is to elucidate the role of emotional expectancy in a self-paced paradigm that unlike reaction time or triggered tasks, does not involve the perception of extra stimuli, such as cues, or additional cognitive processing, such as working memory or discrimination processes. In the current study, the subjects had neither to attend to the stimulus presentation nor to respond to it, but they were instructed to press a key to display an emotional picture on the screen. In other words, there was a temporal concurrence between anticipation and preparation processes because the visual presentation of the stimulus was self-paced, indeed it coincided with the motor response. This methodological issue is very important because it allows the subjects to self-initiate and not just passively receive an affective experience, where the type of emotions and their timing are clearly predictable. This situation is not rare in daily life because we do not just passively experience emotions produced by external events, but we can also deliberately decide to perceive something or not that will affect us emotionally. The use of the MRCPs analysis in an emotional expectancy paradigm might also allow us to shed light on the timing of the activity in the prefrontal cortex (PFC), which was reported to be active in the aforementioned fMRI studies. Indeed, recent studies showed that the PFC activity is detectable using the MRCPs and overlapping in time with the frontal BP component (Berchicci et al., 2012a,b, 2013; Bozzacchi et al., 2012a,b; Sanchez-Lopez et al., 2014). Furthermore, to investigate whether picture processing is affected by expectancy, we adopted a large segmentation including both MRCPs and post-stimulus ERPs. Indeed, we also studied the activity related to the processing of the emotional stimuli measuring the modulation of the P2 and N2 components and the late positive potential (LPP), which is a slow stimulus-related activity reflecting sustained attention to affective contents (Schupp et al., 2000, 2004). This methodological choice was based on the fact that the stimulus-triggered analysis with a baseline shortly before the stimulus onset could mask the pre-stimulus potentials, squeezing them on the 0 μ V activity. Furthermore, the latter method would not be useful for investigating the effects of the pre-stimulus neural adjustments on the modulation of the typical emotional ERPs.

Our hypothesis is that the expectancy of predictable emotions can modulate both MRCPs and post-stimulus brain processing. In particular, in the pre-motor phase, we expect that the more arousing pictures (positive and negative) may modulate both the prefrontal activity and the BP component of the MRCPs more than neutral or scrambled pictures. After the key-press and stimulus presentation, the negative stimuli may further modulate the P2 and N2 components, eliciting enhanced and reduced peak amplitude, respectively (Carretié et al., 2004). Finally, high arousing pictures may lead to larger LPP amplitude, reflecting a sustained attention to emotionally relevant stimuli.

## Materials and methods

### Participants

Fifteen healthy subjects (7 females; mean age = 23.6, *SD* = 4) were recruited from the student population at the Foro Italico University of Rome. The volunteers received an extra credit on the psychology exam for their participation in the experiment. The participants had normal or corrected-to-normal vision and no history of neurological or psychiatric disorders; all of the subjects were right-handed (Edinburgh handedness inventory; Oldfield, 1971). After explanations of the procedures, all of the participants provided written informed consent, approved by the local Ethics Committee.

### Stimuli

Stimuli consisted of 320 affective pictures repeated twice in the course of the experiment for a total of 640 stimulus presentations. Based on their valence and arousal ratings in the International Affective Picture System (IAPS; Lang et al., 1999), we first selected 240 images, equally divided into three emotional categories: positive, negative, and neutral. We adopted the following inclusion criteria: positive and negative pictures had a high arousal rating, but a high and low valence rating, respectively. The neutral pictures were selected based on their medium valence and low arousal rating (see Table 1 for specific ratings of each category). However, to exclude any influence of semantic and autobiographical knowledge on the electrophysiological data, we decided to include a further control condition. For this reason, using CorelDraw™ software, we scrambled each neutral picture to have a scramble category. This approach, already adopted by several other emotional studies (e.g., Schwaninger et al., 2006; McRae et al., 2012), allows the experimenters to keep the perceptual features unaltered, thereby removing the affective content of the pictures. Thus, a total of four emotional categories were employed in the experiment: positive, negative, neutral, and scramble.

**Table 1:** **The affective ratings of the selected IAPS pictures for positive, negative and neutral categories**.

	**Positive**	**Negative**	**Neutral**
Valence: mean (*SD*)	7.13 (0.42)	2.18 (0.5)	5.03 (0.29)
Arousal: mean (*SD*)	6.1 (0.5)	6.4 (0.47)	2.87 (0.42)

### Procedure

During the EEG recording, subjects were comfortably seated in front of a computer screen at a distance of 120 cm. A board was fixed on the armchair allowing the participants to freely push the button panel positioned on it. The fixation point was a yellow circle (0.15 × 0.15° of visual angle) in the center of the computer screen. The participants were asked to alternatively press two keys with the index and middle right fingers in a self-paced rating every 4–5 s (i.e., starting with the left key, they then had to press the right key and then back to the left key, without pressing the same key twice) to display a picture on the screen. Each key-press coincided with the stimulus onset. The experimenter communicated the key-category coupling before each block so that the subjects always knew the affective valence of the stimulus associated with the key-press. The subjects performed 10 trials before starting the experiment to familiarize themselves with the key-press speed. During the experimental session, the experimenter always monitored the interval between stimuli and provided the subject with feedback about his/her speed. Further, the inter-stimulus-interval (ISI) was subsequently calculated to exclude different distributions across blocks. The entire experiment consisted of four blocks, randomly presented and counterbalanced across participants, which were repeated twice. Each block contained 80 pictures, equally divided across each category (i.e., 40 pictures per category) that was associated with a specific key side (see Table 2 for the key-category coupling and ISI values in the four blocks). Each picture lasted 280 ms and each block approximately 6–7 min, automatically ending when all pictures were displayed. The whole experiment lasted 55–60 min.

**Table 2:** **Key-category coupling and inter-stimulus-interval (ISI) in the four experimental blocks**.

**Blocks**	**Key side**		**ISI mean (*SD*)**
	**Left**	**Right**	
1	Positive	Negative	5.26 (0.83)
2	Negative	Neutral	5.23 (0.92)
3	Neutral	Positive	5.2 (1.02)
4	Scramble	Scramble	4.94 (1.06)

*Left and right key sides correspond to the index and middle right finger, respectively. The ISI values are reported in seconds*.

### Electrophysiological recording and data analysis

EEG signals were recorded using the BrainVision™ system (BrainProducts GmbH, Munich, Germany) with 64 electrodes mounted according to the 10–10 International System. All electrodes were referenced to the left mastoid. Horizontal and vertical electrooculograms (EOG) were also recorded using electrodes at the right external canthi and below the left eye, respectively. Electrode impedances were kept below 5 KΩ. The EEG was digitized at 250 Hz, amplified (band-pass of 0.01–80 Hz including a 50 Hz notch-filter) and stored for offline averaging. Artefact rejection was performed prior to signal averaging to discard epochs contaminated by blinks, eye movements or other signals that were detected by an amplitude threshold of ±100 μ V. To investigate the effect of the emotional anticipation on both MRCPs and post-stimulus potentials, the artefact-free signals were segmented based on the key-press that triggered the onset of the visual stimulus and then averaged in 3500 ms epochs (from 2500 before to 1000 ms after the stimulus/movement onset). To further reduce high frequency noise, the averaged signals were low-pass filtered at 25 Hz (slope 24 dB/octave) and baseline corrected from −2500 to −2300 ms. All of the averaged epochs were sorted into four emotional categories: positive, negative, neutral and scramble. For the MRCPs analysis, the mean amplitude of three 500 ms time windows before the key-press (−1500/−1000, −1000/−500 and −500/0 ms) was exported. Based on the scalp topography, we selected the electrodes where the signal was maximal and averaged them to obtain the following pools: left prefrontal (F9, FT9, Fp1), right prefrontal (F10, FT10, Fp2), and occipital (O1, Oz, O2) pools. Following the literature (e.g., Berchicci et al., 2012a), we considered the Cz site for the analysis of amplitude and onset of the BP. The BP amplitude was measured as the mean amplitude in the abovementioned time windows, and the onset latency was calculated as the first deflection larger than twice the absolute value of the baseline mean. For the statistical analysis, separate repeated-measures ANOVAs were performed on the three time windows, with Category and Pool as factors; One-Way ANOVAs were performed on the latency and amplitudes on the Cz site for the BP analysis only.

To investigate how expectancy affects emotional processing, analyses on the post-stimulus event related potentials (ERPs) were also performed. For this purpose, and based on the scalp topographies, the occipital (O2, PO8) and frontal (Fz, FCz) sites were considered for the P2 and N2 components, respectively. The peak amplitudes and latencies of these components were measured for each subject with respect to the −2500 to −2300 ms pre-stimulus baseline and submitted to separate One-Way ANOVAs. Likewise, the LPP was measured on all midline electrodes and divided in two time windows, following recommendations in the literature (e.g., Poli et al., 2007): the LPPa (mean amplitude from 400 to 700 ms after stimulus onset) and the LPPb (mean amplitude from 700 to 1000 ms after stimulus onset). For these components, a 4 × 2 × 8 ANOVA was computed, with Category (positive, negative, neutral and scramble), LPP (LPPa vs. LPPb) and Electrode (AFz, Fz, FCz, Cz, CPz, Pz, POz, and Oz) as factors. Finally, to exclude different distributions of motor presses across blocks, the ISI values were compared by means of repeated measures ANOVA. For all of the mentioned ANOVAs, *post-hoc* comparisons were conducted using Fisher's least significant difference (LSD) test. The overall alpha level was fixed at 0.05.

## Results

**Figure 1:**
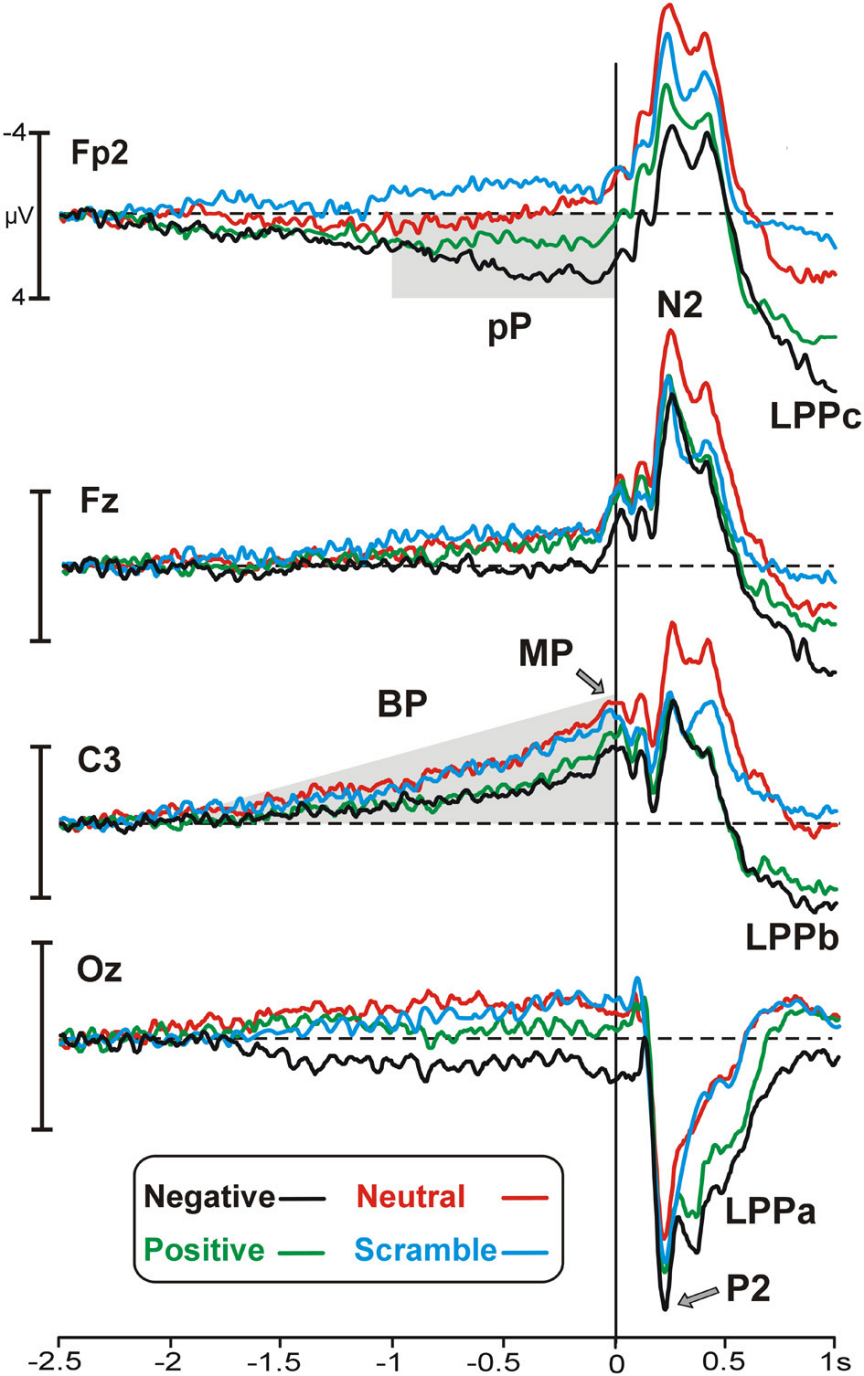
**Grand average waveforms of the emotional categories represented by different colors (specified in the legend) on the most relevant sites**. Time zero corresponds to the key-press and the concomitant stimulus onset. Pp, prefrontal-positivity; BP, Bereitschaftspotential; MP, motor potential; LPP, late positive potential.

**Figure 2:**
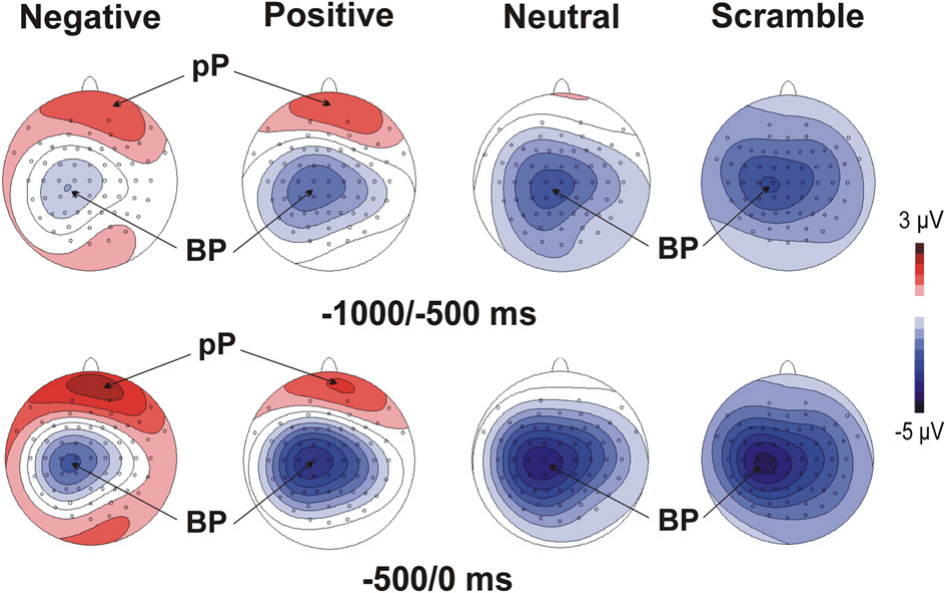
**Scalp topographies (top-flat view) of the grand average of the MRCPs in the four experimental categories**. The maps display the mean amplitude on the scalp in two time windows before the key-press.

Figure 1 illustrates motor/stimulus-related activities in four relevant sites (Fp2, Fz, C3, Oz). Time zero represents the movement onset and the simultaneous stimulus appearance. In all of the emotional categories, the brain potentials started approximately 2 s before the key-press over medial central sites and slowly rose showing the typical negative ramp of the BP. At the same time, a slow rising positivity was also present over prefrontal sites (prefrontal positivity, pP) but only in the two emotional categories (positive and negative). A sustained positive occipital activity started approximately 1500 ms before stimulus onset and lasted until the initiation of the movement in the negative emotional category only. Concomitantly to the key-press, the peak of the motor potential (MP) was prominent over the left central site contralateral to the finger used for all of the categories. The topographical distribution of the pre-motor components described above is shown in Figure 2.

**Figure 3:**
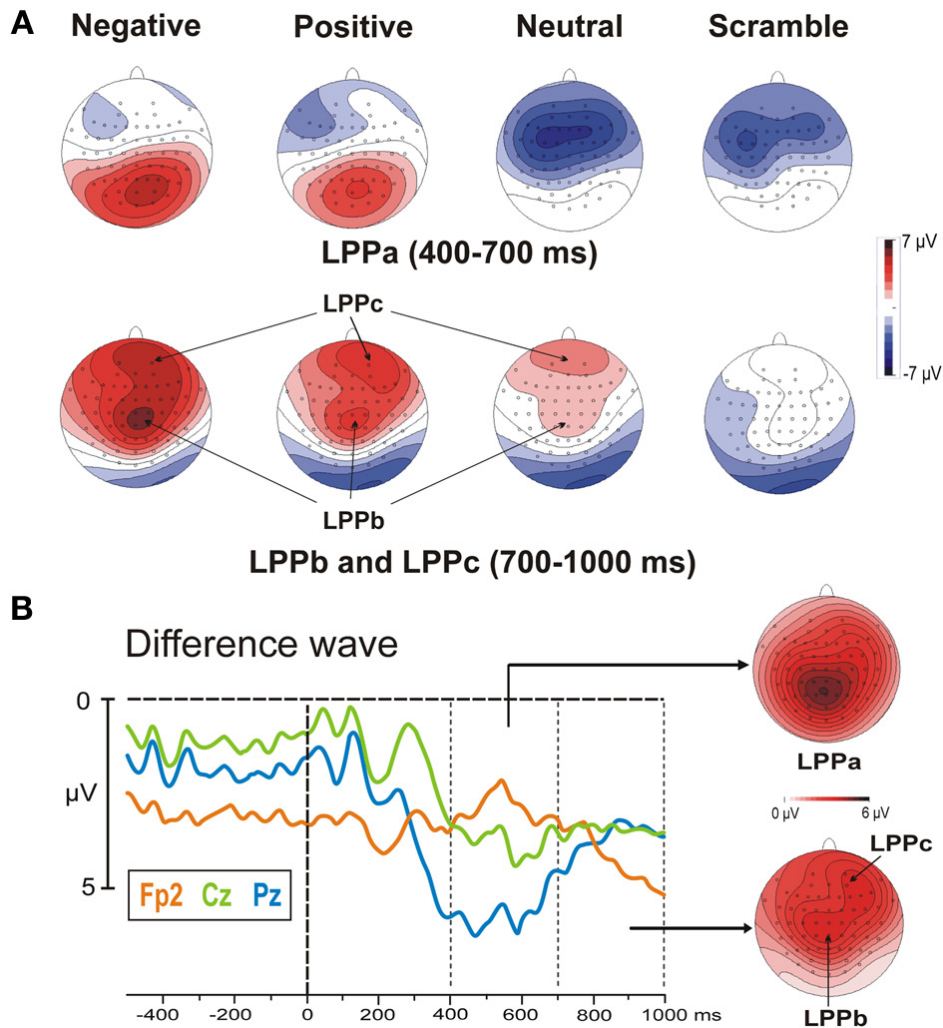
**(A)** Post-stimulus scalp topographical distribution of the LPPa, LPPb, and LPPc in the four experimental categories. It can be observed that negative and positive stimuli elicit larger LPPs. Specifically, the LPPa, LPPb, and LPPc show the maximum activity over posterior, frontal and prefrontal areas, respectively. **(B)** High arousing (positive and negative) minus low arousing (neutral and scramble) categories: differential waves in three representative sites and topographical distribution of the LPPs.

The stimulus onset produced a large P2 at 220 ms over occipital sites and, concomitantly, an N2 over frontal areas, which were more evident in the negative and neutral category, respectively. The late positivities were also present and started over medial parieto-occipital areas (LPPa) at approximately 300 ms and over medial central areas (LPPb) at approximately 400 ms, showing stronger activity for highly arousing categories. Moreover, starting from 500 ms, a third positive activity was also observed in prefrontal areas. We called this potential LPPc because similar to the LPPa and LPPb, it was larger for the more arousing categories. Both the LPPb and LPPc were small (but detectable) in the neutral category and absent in the scramble category. The topographical distribution of the LPPs is shown in Figure 3A, whereas in Figure 3B, the signal is restricted to a smaller time window to show the arousal effect by means of difference wave (high arousing minus low arousing pictures) in different sites.

**Figure 4:**
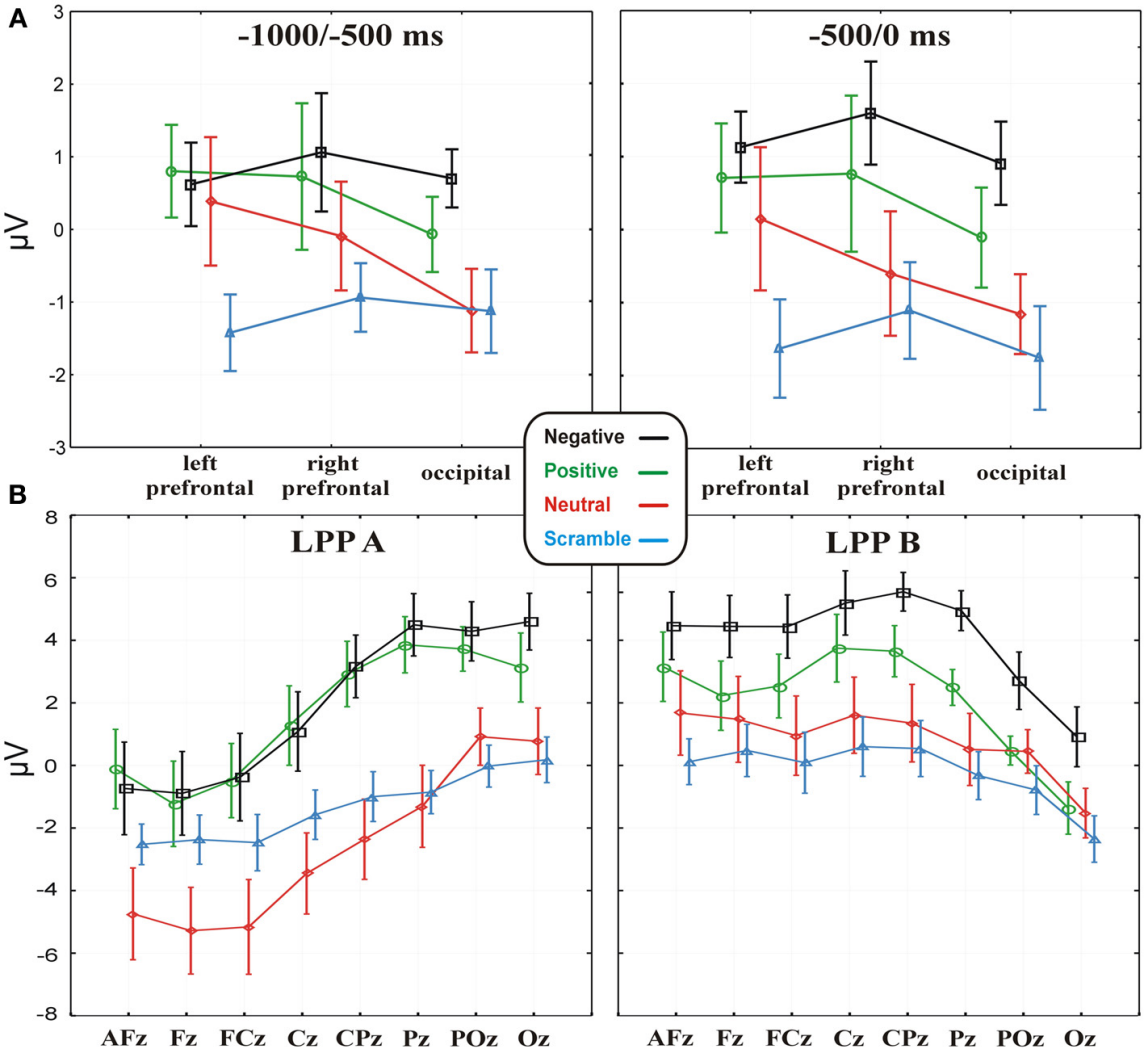
**(A)** Data (values are the mean ± s.e.m.) of the MRCP activities in the four emotional conditions, separately for the −1000 to −500 and −500 to 0 time windows on the three electrode pools. **(B)** Data (values are the mean ± s.e.m.) from the LPPa and LPPb activities in the four experimental conditions on the midline electrodes.

Statistical analysis showed a Category main effect in both the −1000 to −500 [*F*_(3, 42)_ = 3.38, *p* < 0.05] and −500 to 0 [*F*_(3, 42)_ = 4.68, *p* < 0.01] time windows (Figure 4A shows the respective statistical graphs). *Post-hoc* analysis revealed that from 1000 to 500 ms before the key-press, occipital and bilateral prefrontal areas showed a larger positive activity during positive and negative expectancy compared to scramble expectancy (*p* = 0.01 and *p* = 0.005, respectively). Instead, in the −500 to 0 ms time window, the activity at the same electrode pools was greater during positive expectancy compared to scramble expectancy (*p* = 0.01) and during expectancy of negative pictures compared to neutral pictures (*p* < 0.05) and scramble pictures (*p* < 0.001). Neither a Pool main effect nor a Category × Pool interaction was significant. Thus, the more arousing categories showed a large positivity in all of the considered Pools, and no laterality effect emerged on the prefrontal areas. The analyses on the BP mean amplitudes were not significant, whereas the ANOVA on the BP onset showed a significant effect [*F*_(3, 42)_ = 2.87, *p* < 0.05]; the onset of the BP progressively increased across categories from negative (mean = −1.39 s; *SD* = 0.54) to positive (mean = −1.58ṡ; *SD* = 0.51) to neutral (mean = −1.75 s; *SD* = 0.5) to scramble (mean = −1.9 s; *SD* = 0.42). Nevertheless, *post-hoc* analysis revealed a significant difference only between negative and scramble categories (*p* < 0.01). However, the analysis of the latency and amplitude of the P2 and N2 components did not show any significant effects, whereas ANOVA on the LPPs showed a significant Category main effect [*F*_(3, 42)_ = 10.82; *p* < 0.00001], indicating a greater positivity for positive and negative categories compared to neutral and scramble categories. Moreover, the LPP × Electrode interaction effect was also significant [*F*_(7, 98)_ = 69.4; *p* < 0.0001], indicating that the positivity of the LPPa was more pronounced on parietal sites compared to frontal and prefrontal sites, whereas the LPPb showed the opposite trend (see Figure 4B).

No differences emerged between ISI values, such that the motor presses were uniformly distributed across the emotional categories. Furthermore, these data allowed us to exclude possible biases on EEG results.

## Discussion

The present study was designed to investigate how emotions modulate both premotor (MRCPs) and post-stimulus (ERPs) brain activities. To overcome some limitations of externally triggered and reaction time paradigms (which introduce extra brain activity related to external stimuli before response initiation that overlaps and cancels out the premotor activity), we adopted a self-paced paradigm. The results showed that highly arousing stimulus expectancy influences the motor preparation, as shown by the larger MRCP activities over prefrontal and occipital areas compared to the expectancy of neutral and scramble stimuli. The slow positive prefrontal activity started very early, at approximately 2 s before the key-press, together with the BP, and it was prominent during negative picture expectancy. Present findings might appear conflicting with those in which only an enhanced pre-stimulus negativity over central areas was found during negative expectancy (Takeuchi et al., 2005; Poli et al., 2007; Wessa and Flor, 2007). However, this inconsistency could be partly ascribed to the small number of recording electrodes (e.g., Wessa and Flor, 2007), the presence of anticipatory cues (e.g., Poli et al., 2007), or working memory demands (e.g., Carretié et al., 2001) often employed in previous studies. Conversely, in the present study, the subjects themselves created their emotional experience, displaying the pictures by means of key-press at a self-paced rate. Furthermore, because the participants always knew the affective content of the forthcoming picture, they had to focus their attention only on the emotional expectancy and motor preparation. Finally, it is important to remember that the scrambled pictures allowed us to have a condition in which the subjects had merely to prepare a motor response in absence of any emotional expectancy.

To the best of our knowledge, a positive activity over prefrontal and occipital areas has never been found in EEG studies on emotional expectancy, but fMRI and lesion studies could partly explain our results. Indeed, some studies reported increased activation in prefrontal and orbitofrontal regions during expectancy of emotional stimuli (Davidson and Irwin, 1999; Ueda et al., 2003; Nitschke et al., 2006), and Bechara et al. (1994, 1996) repeatedly demonstrated that patients with bilateral lesions of the ventromedial PFC cannot anticipate the future positive or negative consequences of their actions. It was also suggested that the PFC organizes anticipatory behavior in a top-down fashion by activating cortico-cortical and thalamo-cortical loops to sensory and motor areas (Brunia, 1999). Furthermore, the evidence that the perceptual encoding in the visual cortex is modulated by emotional significance of visual stimuli has been reported by fMRI (Lang et al., 1998; Bradley et al., 2003) and ERP (see Olofsson et al., 2008, for a review) studies. Ueda et al. (2003) also observed that the expectancy of and not only the perception of unpleasant stimuli produced a bilateral activation in the visual cortex as well as in prefrontal, amygdala and cingulate regions. Further, the intrinsic relationship between expectancy and motor preparation processes (which overlapped in the present study) was reported by the work of Bermpohl et al. (2006a). They interpreted the emotional expectancy-related activation observed in the parieto-occipital sulcus, supracallosal anterior cingulate cortex (SAC) and cingulated motor area (CMA) as a state of preparedness for action during the expectancy of motivationally relevant stimuli. In brief, they suggested a link between emotional expectancy and motor preparation, even in absence of movements.

It is also likely that the positive activity of the MRCPs over prefrontal and occipital areas reflects an enhanced pre-processing in the to-be-stimulated areas. Our hypothesis is in accord with studies on slow cortical potentials that assumed negative activities, such as the BP, are an index of progressive cortical excitability, reflecting a preparatory state for cerebral processing, whereas the positive activities indicate a decreased excitability, reflecting a greater allocation of perceptual processing resources (Rockstroh et al., 1989; Birbaumer et al., 1990; Schupp et al., 1994). Therefore, the prefrontal and occipital activities may reflect a state of pre-processing of affectively relevant material, anticipating, or facilitating following motivated attentional processes, as reflected by the LPP. In line with the literature (Cuthbert et al., 2000; Schupp et al., 2000, 2003, 2004, 2006; Poli et al., 2007), this latter potential was larger following more arousing stimuli compared to less arousing stimuli, and it was mainly localized over parieto-occipital areas (LPPa). In addition, increased frontal and prefrontal positivity indexed by the LPPb and LPPc (from 700 to 1000 ms after the stimulus onset) was observed. The LPP anteriorization was also found in other studies (Diedrich et al., 1997; Cunningham et al., 2005; Pastor et al., 2008; Gable and Harmon-Jones, 2010), indicating sustained and enhanced attention to emotional stimuli by appetitive and defensive motivational systems. Magnetoencephalographic (MEG) (Moratti et al., 2011) and fMRI (Sabatinelli et al., 2007; Liu et al., 2012) studies have shown that more arousing pictures modulate the LPP and activate an extensive brain network composed of both cortical and subcortical structures, such as the amygdala, parieto-occipital, and PFC. These studies also suggested strong bidirectional influences between frontal and occipito-parietal cortices, leading to top-down and bottom-up processes interacting for emotional stimuli processing. Indeed, as suggested by other authors (Daffner et al., 2003; van de Laar et al., 2004), both prefrontal and parietal lobes contribute to attentional allocation to novel events, but they play different roles; emotional events are more likely processed by prefrontal areas, whereas parietal lobes are mainly involved in the categorization of relevant stimuli. Even if a parietal and frontal LPP have been frequently described in emotional processing investigations, no study reported a prefrontal LPP, as our work does. Thus, it is reasonable to suggest that our paradigm increases the motivated attention to emotional pictures by pre-stimulus processing, as reflected by the pre-motor activities. It has been repeatedly demonstrated that the positive slow waves over frontal and parietal regions between 300 and 900 ms reflect selective attention and working memory processing (Gevins et al., 1995, 1996; Rämä et al., 1995), and as suggested by fMRI studies (e.g., Dolcos et al., 2004), the enhanced PFC activity in emotional evaluation explains the better retention of affective stimuli. Furthermore, as also demonstrated by Bermpohl et al. (2006b), the expectancy of emotional stimuli increased the neural response to the emotional (not neutral) pictures, especially in an emotional network including the MPFC.

Our hypothesis on the affective modulation of the BP was not confirmed; the analyses on the BP have only revealed a later onset during preparation for negative compared to scramble pictures. A delayed BP onset has been previously reported in young people compared to elderly individuals (Berchicci et al., 2012a) and in top-level shooters compared to controls (Di Russo et al., 2005a), reflecting less neuronal recruitment in the supplementary motor cortex (SMA). However, the BP amplitude was not different between emotional categories, most likely because the prefrontal positivity had partially covered the BP activity, leading to a progressively delayed onset for more arousing conditions. In brief, based on these results, it is not possible to confirm emotional modulation of the BP.

At the same time, this study did not reveal affective modulation of the P2-N2 components, which showed the expected emotional modulation trend without reaching statistical significance, as reported elsewhere (e.g., Carretié et al., 2004). The reason may lie in the concomitant occurrence of the re-afferent positivity (RAP), which can partially modify the P2-N2 effect. The cortical generator of the RAP is the somatosensory cortex (Di Russo et al., 2005a,b), thus the cortical distribution of this component is similar to that of the frontal-central N2.

Finally, all of the considered cortical potentials showed the strongest statistical significance in the negative category, especially compared to the scramble one. This finding suggests two main considerations: first, negative stimuli are most likely perceived as more arousing compared to positive stimuli, regardless of IAPS normative ratings (e.g., Poli et al., 2007); and second, scramble stimuli are very useful in emotional studies because of their total lack of affective content. Indeed, although neutral pictures are low arousing and theoretically not related to emotions, they contain faces, objects, and other elements eliciting memories and cognitive evaluations that could be related to affective reactions. A limitation of this study is the absence of a self-report questionnaire on the affective rating, such as the Self-Assessment Manikin (SAM) scale (Bradley and Lang, 1994). Indeed, data about subjective affective ratings could clarify whether the prefrontal and occipital activities during motor preparation are completely related to arousal, irrespective of the valence, or if they are also affected by the negative valence. Unfortunately, it was impossible to use this approach in our protocol because of the high number of stimuli employed. Another limitation of the present study is the time window after the stimulus onset. Indeed, a longer time interval between each key-press could allow a larger segmentation of the signal; we have segmented until 1 s after stimulus onset to avoid the analysis of overlapped segments, but a better LPP modulation could be observed in a larger time window. Finally, because we investigated the MRCPs in a context of self-created emotional experiences, the pre-motor and expectancy activities were obviously overlapped in this design. A paradigm with passive stimuli presentation will be needed to describe the activities more specifically related to the passive expectancy.

## Conclusions

The results of this study show that both MRCPs and post-stimulus processing of highly arousing pictures lead to larger slow positive potentials over anterior and posterior areas, reflecting a state of motivated attention to emotionally relevant stimuli. After picture presentation, the LPPs complex reflected this process, while in the MRCPs time window, a positive potential was observed over prefrontal and occipital regions well before the key-press. These expectancy activities in a context of motor preparation most likely reflects enhanced pre-processing in the to-be-stimulated areas and a state of preparedness for action. We propose that both appetitive and defensive motivational systems could facilitate the forthcoming processing of survival-relevant content, also before the stimulus presentation.

Considering both the emotional-modulation of perceptual encoding in the visual cortex and the role of the PFC in the motivational systems that process the behavioral responses to affective events (Rolls, 2000; LeDoux, 2003), it is likely that the reason why the emotional expectancy is able to modulate the premotor brain activity is to prepare the approach-withdrawal responses to arousing experiences, increasing the probability to do the right thing and, in evolutionary terms, to survive. In conclusion, this study suggests that the response preparation to predictable events leads to specific anticipatory brain adjustments, allowing us to better cope with the subsequent affective experiences.

### Conflict of interest statement

The authors declare that the research was conducted in the absence of any commercial or financial relationships that could be construed as a potential conflict of interest.
